# Polymerizing laminins in development, health, and disease

**DOI:** 10.1016/j.jbc.2024.107429

**Published:** 2024-06-01

**Authors:** Peter D. Yurchenco, Arkadiusz W. Kulczyk

**Affiliations:** 1 Department of Pathology & Laboratory Medicine, Robert Wood Johnson Medical School, Rutgers University, Piscataway, New Jersey, USA; 2 Department of Biochemistry and Microbiology, Institute for Quantitative Biomedicine, Rutgers University, Piscataway, New Jersey, USA

**Keywords:** basement membrane, extracellular matrix, self-assembly, muscular dystrophy, Pierson syndrome, cryo-EM, integrin, dystroglycan, myelination, lamininopathy, polymerizing laminins

## Abstract

Polymerizing laminins are multi-domain basement membrane (BM) glycoproteins that self-assemble into cell-anchored planar lattices to establish the initial BM scaffold. Nidogens, collagen-IV and proteoglycans then bind to the scaffold at different domain loci to create a mature BM. The LN domains of adjacent laminins bind to each other to form a polymer node, while the LG domains attach to cytoskeletal-anchoring integrins and dystroglycan, as well as to sulfatides and heparan sulfates. The polymer node, the repeating unit of the polymer scaffold, is organized into a near-symmetrical triskelion. The structure, recently solved by cryo-electron microscopy in combination with AlphaFold2 modeling and biochemical studies, reveals how the LN surface residues interact with each other and how mutations cause failures of self-assembly in an emerging group of diseases, the LN-lamininopathies, that include LAMA2-related dystrophy and Pierson syndrome.

Basement membranes (BMs) are cell-adherent extracellular matrices that divide tissue compartments and act as solid-phase agonists to affect differentiation and normal tissue functions (1). Their evolution represented a key step in the transition from single-cell organisms to complex differentiated metazoan tissues (2, 3). They exist on the basal surfaces of simple epithelia in sponges, reflecting an early evolutionary role, where they contribute to cell polarization (4). In vertebrates, BMs underlie epithelia, vascular endothelia, and surrounding muscle and fat cells, affecting a variety of inductive and maintenance functions (Fig. 1).

**Figure 1:**
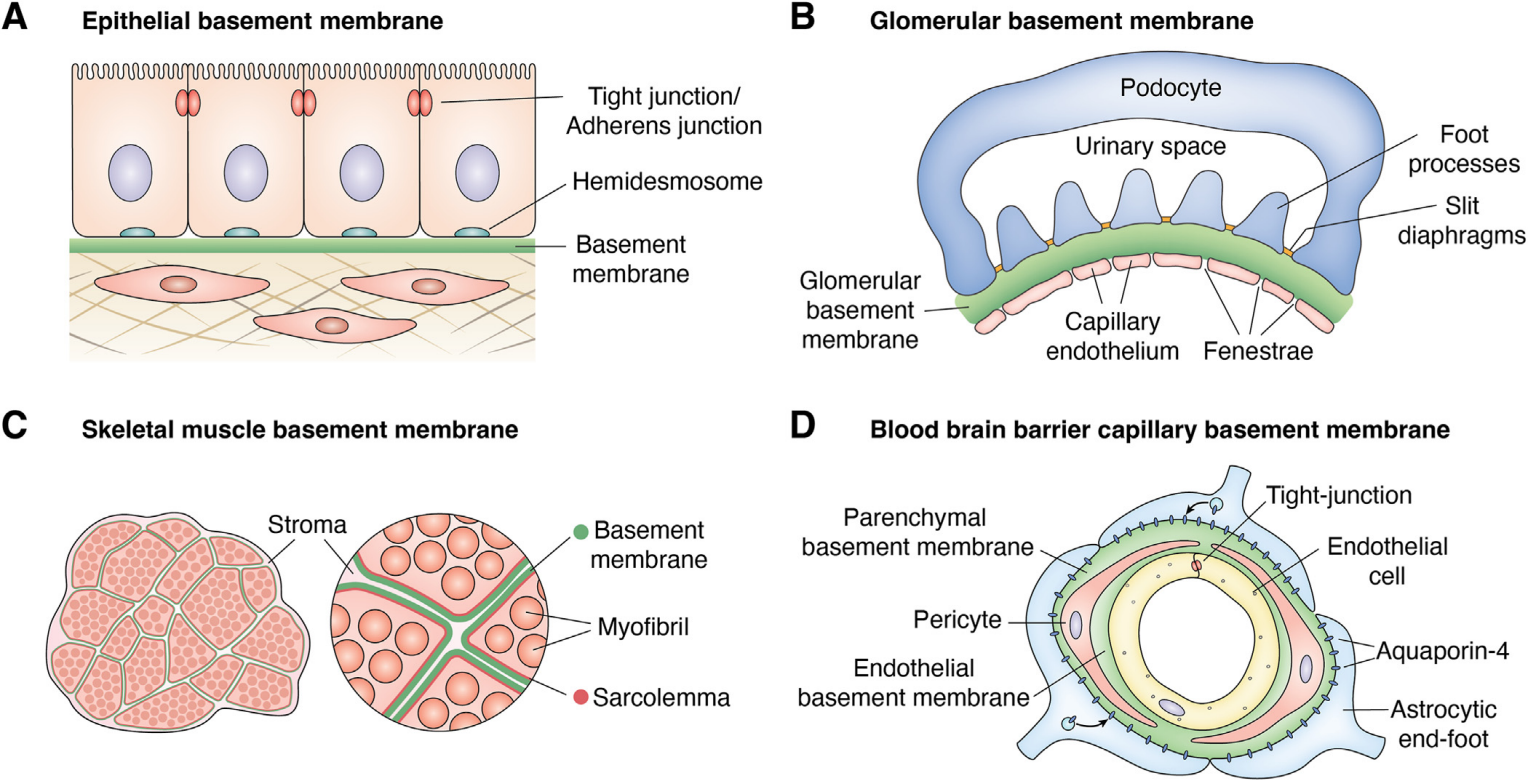
**Basement membranes.** These ECMs are found anchored to cell surfaces of epithelial, endothelial, muscle, and fat cells, but generally not fibroblasts, cartilage, or osteocytes. *A*, epithelial BMs are typically thin (<100 nm) and are adjacent to the stroma. They are invested with α5 and α3 laminins. *B*, the mature glomerular basement membrane (300–350 nm in humans) is a filtration barrier enriched in Lm521 that results from the fusion of capillary endothelial and podocyte BMs. The endothelium is fenestrated while the podocyte foot processes are separated by slit diaphragms, leaving the GBM as the only continuous barrier between the blood and urinary space. *C*, mature skeletal myofibers (Lm211) are each coated with a thin BM along their length with a sparse collagenous stroma between each BM. Specialized BMs are found at the neuromuscular junctions (Lm221, 421) and myotendinous junctions. *D*, capillary BMs of the blood–brain barrier differs from typical capillary BMs in that they are double separated by pericytes. Components are generated by endothelial, pericyte, and glial cell foot processes such that the endothelial BM contains Lm511 and 411 while the glial BM contains Lm111 and 211.

Members of the laminin family are critical components of BMs. They initiate assembly and are responsible for a major component of BM receptor-mediated signal transduction (1, 5). Loss or alteration of laminins results in failures of embryonic development and diseases manifesting in defects of the kidney, eye, muscle, peripheral nerve, brain, and skin. An understanding of the molecular etiology and pathogenesis of these diseases requires an understanding of laminin structure–function relationships. This knowledge may help enable the engineering of repairs of defective BMs in diseases and the designing of biomimetic BMs for tissue implants (6).

## Basement membrane components and receptors

### Basement membrane composition

Proteomics has revealed that BMs contain multiple macromolecular components (7). However, the major structural elements shared by the different BMs and needed for assembly are few, consisting of laminins, type IV collagens, nidogens, and the heparan sulfate (HS) proteoglycans perlecan and agrin (8). The formation of BMs is largely one of the regulated self-assembly initiated by BM protomers secreted into cell-adjacent diffusion-limited spaces. Assembly requires binding among these protomers on a cell surface to form a solid-phase and ultimately insoluble planar extracellular matrix (ECM). Linkage of the matrix to transmembrane receptors enables anchorage to the cytoskeleton. Signal transduction by the ECM is mediated through the receptors and modulated by their specificities, affinities, and resistance to cell surface deformation (9).

### Laminins

**Figure 2:**
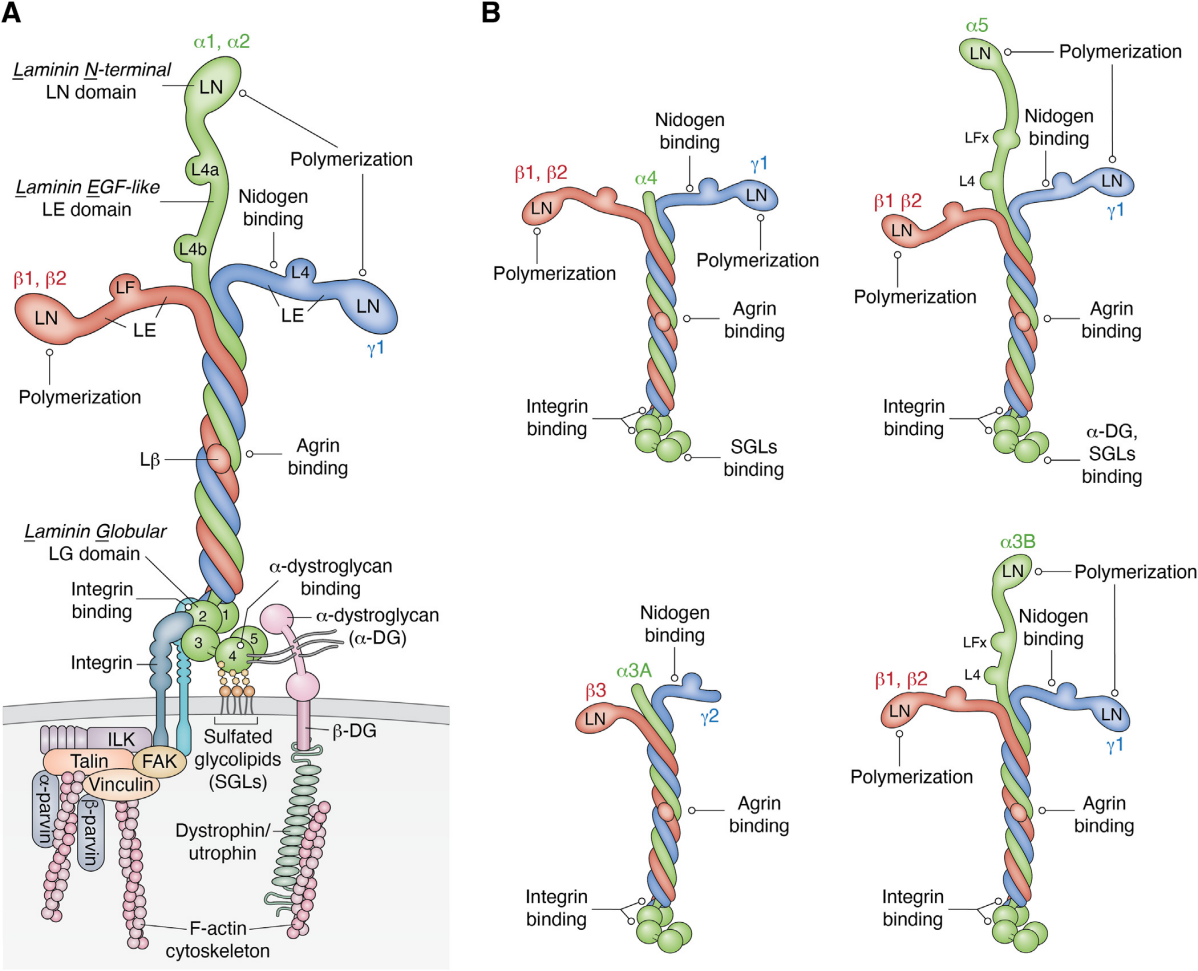
**Family of laminins with γ1 or γ2 subunits.***A*, laminins 111, 211, 121, and 221 share the same domain structure with similar binding domains. Of these, the LN domains, globular in shape, mediate polymerization. The rod-like LE domains, typically containing eight cysteines that are internally paired, act as spacers. The γ1LEb3 domain mediates nidogen-binding. A three-chain α-helical coiled coil that forms the long arm and contains a binding site for agrin. The LG domains are arranged into a cluster of three proximal domains (LG1-3) connected by a flexible linker to two distal domains (LG4-5). LG1-3 and distal coiled-coil mediate the major integrin while LG4 (and LG1-3) mediate α-dystroglycan (αDG) receptor binding. Laminin-receptor complexes are shown for α1/2 laminins. The LG domains of laminins provide the principal ligands for receptor interactions. LG4 binds to sulfated glycolipids such as sulfatides and to heparin/heparan sulfates, and α-dystroglycan (αDG). Secondary αDG binding sites are also present in LG3 (both Lmα1 and Lmα2). Integrins (α6β1, α7β1) bind to a complex of LG1-3 that requires the terminus of the colied-coil domain. The cytoplasmic portion of integrins binds to adaptor proteins that in turn bind to F-actin. *B*, laminins 511 and 521 differ in their longer α-short arm containing a pair of RGD integrin-binding sequences for αv integrins, a partially different repertoire of integrins, and a binding site for the Lutheran antigen/BCAM receptor. Lm411 and Lm511 have truncated α-short arms (preventing polymerization) and do not bind to αDG. They bind to α6β1 and α7β1 integrins, weakly in the case of Laminin-411. Laminin-421 also binds to CD146/MCAM, affecting cell migration. Laminin-3A32 (Lm3A32) is a non-polymerizing largely hemidesmosome-associated epithelial laminin with strong integrin interactions. Maturation cleavages occur in the β3 and α3 short arms such that it assumes a rod-like appearance. Laminin-3B11 is an α3 splice variant with a polymerizing LN domain that has been reported to be expressed in vasculature and other sites. While it is unclear what biological role it might play, they may be unique in their integrin integrin-binding repertoire in the absence of αDG binding. γ3-laminins (not illustrated), similar in domain organization to γ1, lack a critical C-terminal sequence required for integrin binding.

Laminins, the first one described by Timpl *et al.* (10, 11), are a family of large multidomain glycoproteins. Each laminin isoform (Fig. 2) consists of an α, β, and γ subunit joined through a long coiled-coil (12). Of 11 identified mammalian subunits (α1-5, β1-3, γ1-3) and a major splice variant (α3B), 16 isoforms have been shown to assemble into heterotrimers (13). For each laminin, we now employ a simplified code to indicate its subunit composition, for example, Lm111 for α1β1γ1 and Lm211 for α2β1γ1 (12). These laminins differ in their ability to polymerize and to interact with receptors (Table 1). Diverse tissue BMs differ in their laminin composition with most containing more than a single isoform.

**Table 1:** Laminin binding activities

Laminin	Polymer	LG-integrin, other	αDG^b^	Other receptors/Adhesion molecules	BM binding	Tissue examples	References
Lm111 Lm121	yes yes^a^	α6β1,α7X2β1, α6β4 (weak) same, ↑ affinity/adhesion	LG4 (strong); LG 1–3 (weak)	Sulfatide, H/HS, Gpr126^a^	Nd, agrin, HS	Kidney, BBB, embryonic BMs	(5, 17, 60, 62, 233, 234, 235, 236)
Lm211 Lm221	yes yes^a^	α7X1β1, α7X2β1 ↑ affinity/adhesion^a^	LG5, 1–3 (strong)	Sulfatide, H/HS, Gpr126	Nd, agrin, HS	Muscle, p. nerve SC, BBB Perineurium	(5, 14, 17, 20, 236)
Lm3A32 Lm3B11	no yes	α3β1,α6β1,α6β4	no no			Epithelia, p. nerve, small blood vessels	(5, 14, 31, 236)
Lm411 Lm421	no no	α6β1 (weak), α7X1β1 (weak) ↑ integrin adhesion	no no	Sulfatide, H/HS MCAM, sulfatide		Kidney, p. nerve, vasculature NMJ	(5, 14, 60, 236)
Lm511 Lm521	yes yes	α3β1,α6β1,α6β4,α7X1β1	LG4-5, (weak) LG1-3 (v. weak)	αv in αL4, BCAM in LG αv in αL4, BCAM in LG	Nd, agrin, HS	Epithelia GBM, eye, NMJ	(5, 31, 65, 236)
Lm213 Lm523	yes^a^ yes^a^	No integrin binding No integrin binding	LG4, 1–3 weak		Uncertain	Heart, other Epithelium, brain	(19)

Abbreviations: αDG, α-dystroglycan; BBB, blood-brain barrier; GBM, glomerular basement membrane; H/HS, heparin/heparan sulfate; Itg., integrin; Nd, nidogen; MCAM, melanoma cell adhesion molecule, CD146; NMJ, neuromuscular junction; p.nerve, peripheral nerve.

a Predicted activity.

b αDG-laminin K_D_s: α1,∼20 nM; α2, 43 nM; α4, ≥1 μM; α5, 150 nM.

Laminins with “full-length” subunits (α1, α2, α3B, α5, β1, β2, γ1, γ3) have a cross-like appearance with three short arms each tipped by an LN domain and one long arm. The short arms mediate polymerization and nidogen binding. On the other hand, laminins containing the α3A, α4 and γ2 subunits have truncated or absent short arms and lack the LN domains required for polymerization (14). The α-subunits of all laminins extend beyond the end of the coiled-coil with the terminal moiety organized into five globular (LG) domains. These domains mediate binding to integrin and dystroglycan receptors as well as to cell surface components that include sulfated-glycolipids and heparin/heparan sulfates (15, 16, 17, 18). The γ3 subunit, similar in domain organization to γ1, only assembles into Lm213 and Lm523, both predicted to polymerize (13). The γ3 subunit is unique in that it lacks a γ1 glutamic acid near the C-terminus essential for LG domain-mediated integrin binding (19). Further laminin diversity is created by proteolytic processing of short arms and LG domains. Lm332 is extensively truncated while Lm211 and 221 become cleaved within the LG3 domain, decreasing heparin binding (20). It has been proposed that the proteolytic modifications represent regulatory processing to alter signaling functions (20, 21, 22).

Of the truncated laminins, Lm3A32 is a non-polymerizing epithelial laminin containing the short α3A splice variant subunit. It is secreted as a 460 kDa precursor that is enzymatically cleaved in the α3 and β2 chains to form a functional rod-like protein (23, 24). Lm3A32 is an important component of the dermal-epidermal linkage, forming a bridge between basal keratinocytes and the underlying dermis (25). It binds to collagen-VII of anchoring fibrils on the dermal side and to integrin α6β4 at keratinocyte hemidesmosomes (26, 27). Hemidesmosomes, in turn, are organized protein complexes linked to keratin intermediate filaments. Lm3A32 is not required for BM assembly (28). Null mutations in the Lm332 subunits causes the lethal disease Herlitz-type junctional epidermolysis bullosa (JEB) while missense mutations can result in a milder form of JEB (29). The longer Lm α3B splice variant, found in the developing brain and other organs, possesses an LN domain followed by rod-like LE repeats (30). When joined with the β1 (or β2) and γ1 subunits, it is a polymerizing laminin with higher affinity self-interactions (31). Miyazaki and colleagues reported the presence of Lm3B11 as a third α-subunit in vasculature, accompanying α4 and α5 (32). Further, they reported that the 3B subunit, while present in normal vasculature, was absent in neoplastic vasculature (33). If the integrin binding of Lm3B11 is similar to that of Lm3A32, then this laminin is unique in that it would possess high-affinity polymerizing and high-affinity receptor binding characteristics and thus might have potent signaling characteristics.

### Integrins and laminins

Integrins are transmembrane heterodimeric receptors that mediate signaling initiated by ligand binding (34). A key property of integrins is that of bi-directional signaling in which integrins undergo conformational transitions between an inactive and active state initiated by cytokines and other agents (35). The mechanism of signaling, studied largely in non-BM ECM interactions, is thought to apply to BMs as well. Integrins connect the ECM with the actin cytoskeleton and transduce mechanical forces generated by actin flow and myosin II in what has been described as a molecular clutch (36). It has been proposed that the glycocalyx layer on cell surfaces, thinner than the height of active integrins, exerts steric and osmotic repulsion to the ECM such that the glycocalyx must be mechanically compressed upon integrin-ligand binding (36). Such compression allows the complexes to sense the pulling force resulting in integrin activation and signaling. Mechanical integrin stretch activates focal adhesion kinase (FAK) and Src family kinases. The maturation of focal adhesions (FAs) through recruitment of the adaptor protein vinculin and FAK in response to integrin activation is mediated in part by phosphorylation of non-muscle myosin II (37, 38). Cells are thought to adopt their signaling in response to ECM rigidity to control proliferation and differentiation, *for example*, substrate stiffness/elasticity predisposes stem cells to pursue different cell fates (9). Drivers of BM stiffness are the laminin and collagen-IV viscoelastic polymers (39), the latter of which is important to achieve higher degrees of stiffness through the formation of covalent crosslinks. Laminin polymer stiffness depends upon the laminin polymer density (40). In mixed laminin BMs (*e.g.*, vasculature), non-polymerizing laminin (*e.g.*, Lm411) admixed with polymerizing laminins (*e.g.*, Lm511) is expected to reduce polymer stiffness. Further, stiffness, with alterations in signaling, can be decreased by netrin-4 (40) and perhaps by other laminin-binding proteins as well. The establishment of linkages between the BM and cytoskeleton through receptors is crucial for epithelial polarization (41, 42).

Laminins bind to integrins α1β1, α2β1, α3β1, α6β1, α7β1, α6β4, and αvβ3/5, the last to α5-laminins (15). Key β1-integrin interactions are between integrins α3β1, α6β1 and α7β1 and the LG1-3/coiled-coil complexes of Lm111/121, Lm211/221, Lm332, and Lm511/521. Laminins 332, 511, and 521, enriched in epithelia, also interact with integrin α6β4, the latter at sites of hemidesmosome complexes that connect the keratin cytoskeleton to the BM (27). Integrins α1β1 and α2β1 (primarily collagen receptors) and integrin α3β1 were reported to bind to the LN domains of the laminin α1, α2, and a5 subunits based on cell adhesion observed with isolated laminin fragments; however, these interactions were not detected with intact laminins and likely represent minor interactions (43, 44, 45, 46). Finally, the two RGD sequences in the L4 domain of Lm511/521, located in the α5-short arm, mediate substantial interaction contributions through αvβ3 and αvβ5 integrins (46, 47).

Early studies of integrin-laminin interactions revealed a conformation-dependent requirement of the terminal coiled-coil domain and LG1-3 (48, 49). Subsequent studies identified a requirement of a γ1-glutamic acid three residues upstream of the C-terminus for binding as well as a modulating affinity effect mediated by the terminal segment of the β1 subunit (50, 51). Most recently the structures of the quaternary integrin α6β1/Lm511 complex were solved by cryo-electron microscopy (cryo-EM) and X-ray crystallography (52). The study, detailed below in Structural probing of laminin interactions with BM components, represented a tour de force that required the use of stabilizing antibody fragments. The complexity of the interaction not only reveals the requirement for more than single interacting domains but also poses a challenge for attempts to mimic the biologically important high-affinity receptor interaction with small proteins or synthetic peptides for the development of synthetic BMs.

### LG-dystroglycan interaction

Dystroglycan (DG) is widely expressed as part of a transmembrane glycoprotein receptor complex important for BM interactions in skeletal muscle, peripheral nerve, brain, and eye (53, 54, 55). An initial single DG transcript is cleaved into an N-terminal moiety (αDG) and a C-terminal moiety transmembrane domain (βDG) (56). αDG, in turn, can be cleaved by cells releasing αDG-N (57, 58, 59). A unique αDG glycan, matriglycan, located in the neck-like region of αDG, binds to the LG domains of α1, α2 and α5 laminins, agrins, and perlecan (60, 61, 62, 63, 64, 65). Matriglycan consists of a repeating xylose-glucuronate discaccharide bound to αDG through an O-mannosyl linkage (66). Synthesis of this adduct (reviewed in detail in (67)), starts with O-mannosylation of αDG by POMT1/POMT2, addition of GalNAcβ1-3 and GlcNAcβ1-4 by POMGNT2 and β3GALNT2, phosphorylation of the mannose by POMK, addition of a ribitol-phosphate moiety by FKTN and FKRP, addition of xylose and glucuronate saccharides to ribitol-phosphate by TMEM5 and β4GAT1, and completion by addition of repeating [3GlcAß1,3Xyla1-] disaccharides by the bifunctional glycosyltransferase LARGE (16).

Defects in glycosylation that prevent LG-binding cause dystroglycanopathies affecting muscle with or without brain and ocular abnormalities (muscle-eye-brain disease, Fukuyama CMD, and Walker-Warburg syndrome). βDG binds to cytoskeletal dystrophin that in turn is linked to the actin cytoskeleton. βDG is part of the dystrophin–glycoprotein complex consisting of the muscle of Dp427-M, DG, sarcoglycans, sarcospan, dystrobevins, and syntrophins (68, 69). It is thought that the dystrophin–glycoprotein complex also acts as a mechano-transducer. The crystal structure of matriglycan complexed with the LG domains from laminin α2 was recently determined and it is described ahead.

### Specialized laminin receptors

Other cell surface-exposed laminin-binding receptors, apparently not required for primary cell anchorage, include Lutheran antigen/B-CAM (Lu/B-CAM, CD239), CD146 (MCAM), and the G-coupled protein receptor 126 (Gpr126, VIGR, DREG). Lu/B-CAMs are erythroid immunoglobulin superfamily receptors specific for the LG domains of α5-laminins found on red blood cells, some epithelia, and other cell types (70). Their functions are uncertain. CD146 is a transmembrane receptor of vascular endothelia with a soluble form that binds to α4-laminins, in particular Lm421 (71, 72). It is reported to promote the migration of tumor cells in concert with integrin α6β1. Gpr126 is a laminin-binding protein that is required for Schwann cell development and radial axonal sorting leading to myelination (73, 74).

### Laminin self-assembly

**Figure 3:**
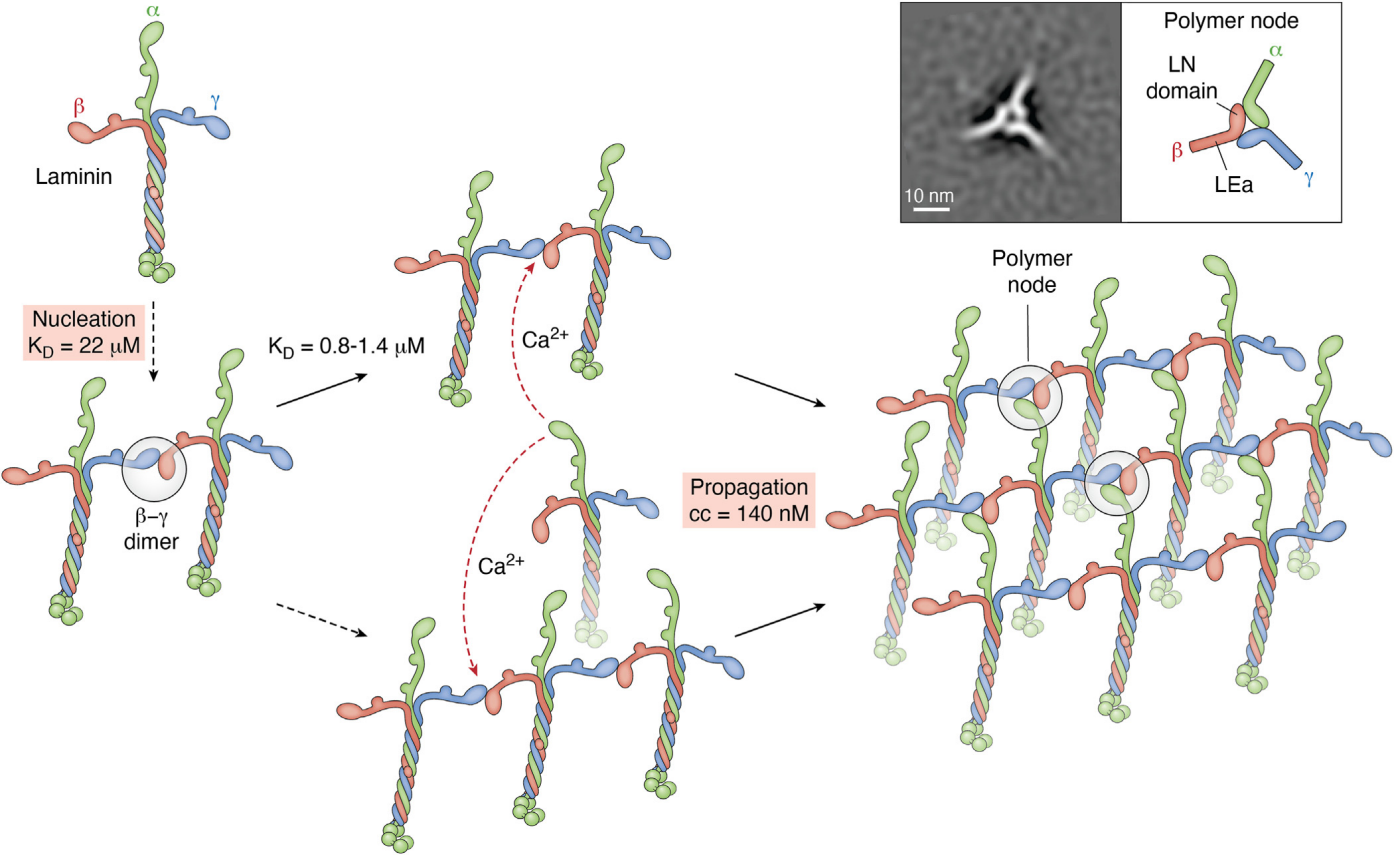
**Laminin polymerization.** The laminin first assembles into a β-γ dimer through weak LN domain interactions. Adding a third laminin through another β-γ bond is possible. Importantly, the α-LN domain of free laminins binds to the available β-γ intermediate complexes through a higher affinity calcium-dependent interaction, completing the formation of polymer nodes. An image-averaged negative stain of a polymer node is shown in the *inset*.

Laminin polymerization was discovered from the aggregation behavior of native Lm111 (75). The process, one of self-assembly, was found to be reversible and concentration-dependent with cooperative nucleation-propagation characteristics (Fig. 3). Electron microscopy of rotary shadowed platinum/carbon (Pt/C) replicas of self-assembled laminin solutions revealed that linear oligomers formed in the presence of EDTA, while further assembly into larger complexes required calcium, suggesting a two-step process. The polymers were visualized by high-angle thin Pt/C replicas following deep etching as a fine meshwork of interconnecting struts (76). E4 and E1’, proteolytic fragments collectively containing all short arm domains, reversibly bound together while other fragments did not, indicating a three arm polymerization model requiring all three, *that is*, α1, β1, and γ1 LN domains (77). Subsequent analyses with recombinant laminins and their fragments confirmed that polymers only formed with laminins possessing the three different subunit LN domains (78, 79). Laminin assembly on cell surfaces, on the other hand, required laminin LG domains. Interestingly, only LG-containing laminins accumulated on cells when mixed with laminins lacking LG domains, *that is*, the polymer will not integrate LG-less laminins on cells. One gap in our knowledge is whether a given laminin can simultaneously bind to integrin and αDG.

Further insights into the laminin polymer depended upon the solving of the structure of the LN domains and their adjacent LE repeats by crystallography (80, 81), analysis of the binding of mutagenized fragments (79, 82), image-averaging of negatively-stained complexes (82, 83), and finally cryo-electron microcopy, as described ahead (83). A size-exclusion chromatography (SEC) analysis of the wild-type fragments revealed that ternary node complex formation occurred in two steps (79, 82). The first step is the dimerization of the β- and γ-LN domains through a low-affinity interaction (K_D_ = 14–22 μM) in which dimers serve as obligate substrates for addition of the α-LN domain through a higher affinity calcium-dependent interaction (K_D_ = 0.8–1.4 μM) in a second step. Homologous interacting faces are present on the α1, β1, and γ1 LN domains with glycans on the opposite face (80, 81). Image averaging of the polymer node complex revealed a heel-to-toe organization of the LN domains with angled outward projections of the LEa stem-like domains and a small central hole. The identities of the toe-heel binding were determined by SEC using LN-LEa segments bearing point-mutations (82). Dimer-blocking mutations were confined to the γ1-toe and the β1-heel, whereas the trimer-only-blocking mutations mapped to the γ1-heel, β1-toe, and the α1-toe and heel. Thus, in the polymer node the γ1-toe pairs with the β1-heel, the β1-toe pairs with the α1-heel, and the α1-toe pairs with the γ1-heel.

## A general model of laminin and BM assembly

Laminins are essential for vertebrate BM assembly (84, 85, 86). This is likely a consequence of their unique repertoire of direct and indirect binding interactions. Assembly occurs only on adhesive (“cognate”) cell surfaces (18). While BMs are found on epithelia, endothelia and muscle fibers and fat cells, most fibroblasts do not have BMs, even though they can be found adjacent to epithelial or other cell BMs (18). This implies that laminin assembly, the initiator of BM assembly, occurs only on those cell surfaces whose receptors or other adhesion molecules specifically bind to laminins. The primary laminin ligands that interact with the cell surface for assembly and anchorage are the LG domains (1, 15, 66).

**Figure 4:**
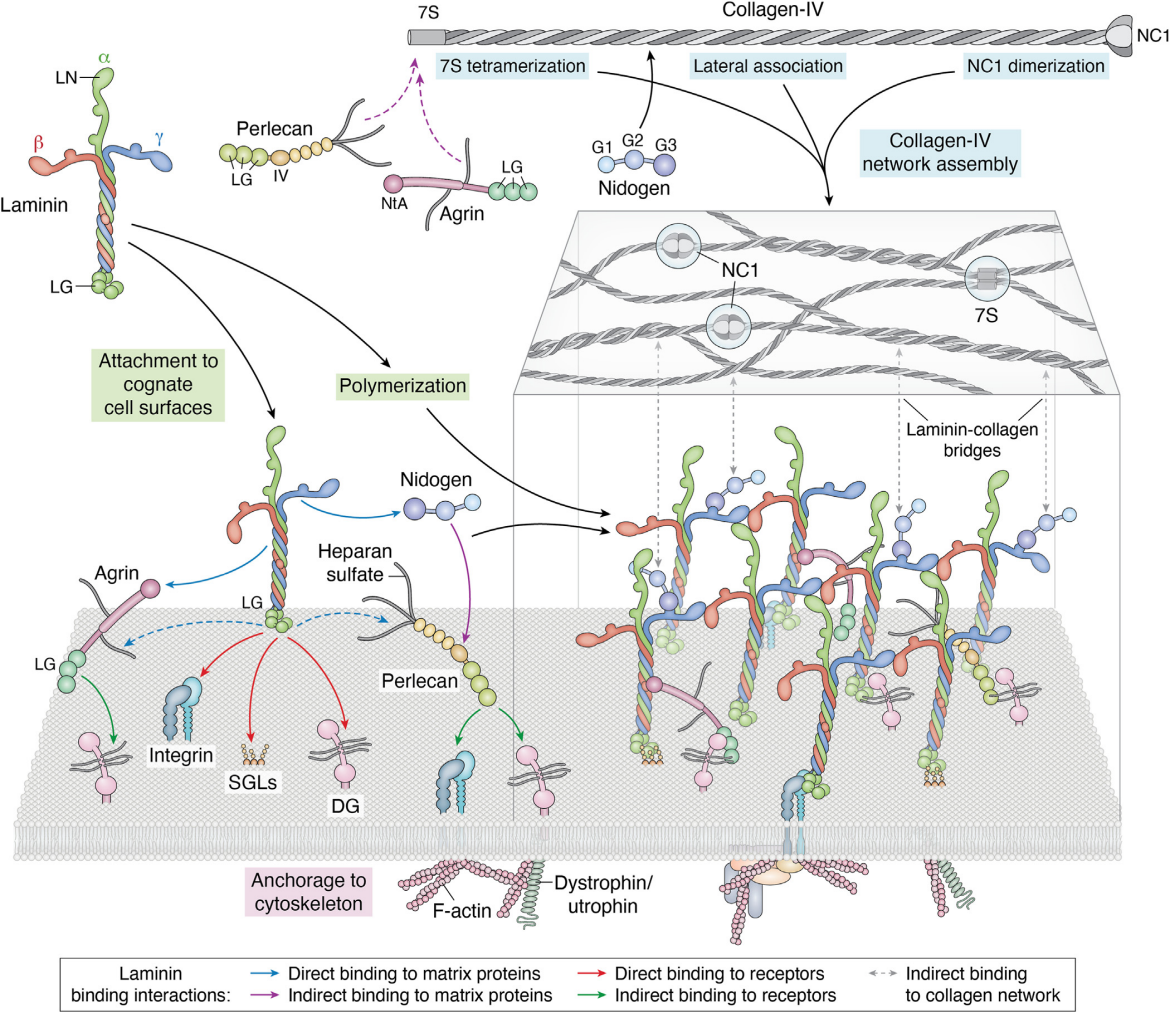
**Basement membrane assembly through polymerizing laminin.** In neuromuscular tissues, Lm211 attaches to the cognate cell surface as it polymerizes. Laminin can form an initial scaffolding by binding to SGLs (sulfatides in Schwann cells), and to laminin-class integrin and α/β-DG receptors. The receptors, in turn, are anchored to the cytoskeleton. Nidogens-1 and -2 and heparan sulfate (HS) chains (when sufficiently sulfated) bind to laminin (Lm) and to collagen-IV, acting as bridges, with the collagen polymerizing through 7S, NC1, and lateral associations to form a second network. Agrin binds through its N-terminal globular domain (NtA) to the laminin coiled-coil and to DG through its C-terminal moiety and perlecan (Perl) domain IV binds to the nidogen G2 domain. Thus, like nidogen, proteoglycan HS chains are thought to form a bridge between the laminin and collagen polymers.

Laminin domain-dependent self-assembly on cultured neuromuscular cell surfaces and its recruitment of nidogen-1, perlecan, and collagen-IV has been modeled in tissue culture and structural studies to gain a better mechanistic understanding of the general process (76, 78, 87, 88, 89, 90, 91, 92, 93, 94, 95, 96, 97, 98, 99). During initial steps, locally secreted laminins adhere to selective cell surfaces, sometimes initially to galactosyl-sulfatides (18), and become anchored to the cytoskeleton through transmembrane receptor complexes (18, 100, 101, 102, 103, 104, 105, 106) (Fig. 4). In cultured Schwann cells and fibroblasts it was found that enzymatic desulfation of galactosyl-sulfatide prevented laminin cell surface accumulation while sulfatide-loading of fibroblasts enabled laminin accumulation (18). However, sulfatides are outer leaflet plasma membrane glycolipids that seem unlikely to mediate signaling. Instead, the integrin and/or DG transmembrane receptor complexes are needed for establishment of cell polarity and BM-cell signal transduction. Polymerizing laminins are normally found in all or nearly all BMs. While non-polymerizing laminins can initiate BM assembly (as seen in muscle and peripheral nerve BMs of Lama2^−/−^ mice), the resulting BMs in muscle and peripheral nerve exhibit low BM density accompanied by functional deficiencies. It is thought that cell attachment increases the laminin on the cell surface above its solution critical concentration of polymerization, facilitating assembly of a two-dimensional planar polymer that constitutes a provisional BM matrix (107).

As assembly proceeds, the laminins recruit other BM proteins by binding to (i) the C-terminal G3 propeller domain of nidogens (secreted by fibroblasts) through Lmγ1-LEb3 in the short arm (108, 109, 110), (ii) the N-terminal NtA domain of the heparan sulfate proteoglycan (HSPG) agrin through the laminin coiled-coil (108, 111), and (iii) heparan sulfates through the laminin LG domains (17, 60, 62, 112). Indirect laminin interactions are: (i) the nidogen G2 domain binds to collagen-IV (108, 113) and HSPG perlecan core protein (114, 115), (ii) perlecan core LG domains bind to αDG and integrin (116), and (iii) agrin core LG domains bind to αDG. The HS chains, as found in agrin, perlecan and other proteoglycans, exhibit heterogeneity in their N- and O- sulfation and binding affinities that depends on the synthesizing cell type (117, 118, 119). In addition to providing attachment and activation sites for growth factors and morphogens, these HS chains have the potential to stabilize BMs and link laminin and collagen-IV polymers by binding to laminin LG domains, collagen-IV 7S, and other protein domains (120, 121, 122).

Collagen-IV monomers dimerize through their NC1 domains (98, 123) and polymerize by the formation of N-terminal (7S) tetrameric bonds and lateral associations that result in triple helical supercoils (93, 95, 124). NC1 dimer assembly is chloride ion-dependent in most species with bond stabilization through the formation of sulfilimine crosslinks (98, 123, 125). The collagen polymer is covalently crosslinked at its N- and C-terminal domains, stabilizing the nascent laminin ECM (96, 98, 101, 126).

Non-neural and neural agrin can form collateral linkages with αDG and integrins, acting to increase cytoskeletal anchorage and, along with perlecan, present heparan sulfates for growth factor and morphogen binding and activation (63, 111, 114, 115, 127, 128, 129, 130, 131). Studies in the invertebrates *D. melanogaster* and *Caenorhabditis elegans* also support a laminin-based mode of BM assembly (132, 133, 134, 135). An exception of the rule might exist in *C. elegans* pharynx, in which there appears to be laminin-independent integrin-dependent assembly of collagen-IV; however, assembly on a pre-existing matrix has not been ruled out (133).

### Netrin-4 can regulate matrix stiffness by depolymerization of laminins

Netrins are well-known developmental guidance factors of axons and other cell types. Each possesses an N-terminal LN-like domain, EGF-like domains, and a unique C-terminal domain (136). Because of their LN domain homologies and detection in BMs, it has been hypothesized that they bind to laminin polymer nodes. However, this interaction has only been established for netrin-4, a netrin with LN homology to the Lmβ1 subunit (137, 138). Netrin-4 binds strongly to the Lmγ1 LN domain such that it readily out-competes Lmβ1 LN (and does not support Lmα1 binding), thus inhibiting polymerization. The interaction disrupts the polymer continuity in a concentration-dependent tunable manner, reducing stiffness (40). This structural alternation has been associated with the metastatic potential of tumor cells. LaNtα31 is a short Lmα3B splice-variant, similar in structural organization to classical netrins, with wide epithelial and vascular endothelial distributions, affecting cell adhesion/migration (139, 140). Given that the Lmα3 LN domain can join with the β1 and γ1 LN domains, it should be able inhibit laminin polymerization through competition (31). The function may simply be to deliver a novel receptor ligand to BMs. As a regulator of polymer stiffness, however, the affinity is likely much less than that of netrin-4, rendering it less potent.

### Functional redundancy and assembly heterogeneity

The basis of laminin to collagen-IV linkage has been controversial. Timpl and colleagues published that nidogen-1 binds strongly (K_D_ < 1 nM) both to laminin-111 and to collagen-IV in solution to form a ternary complex (108) and also described a nidogen-2 homolog with a similar tissue distribution but weaker laminin-binding and lower expression (141). They proposed that nidogens serve a universal role in linking the laminin and collagen-IV polymers (142, 143). However, Bader *et al.* found that while the mouse nidogen-1/nidogen-2 double-knockout resulted in perinatal lethality with various BM defects, the knockout failed to prevent the embryonic formation of BMs containing laminins, collagen-IV, and perlecan. These findings, along with related observations in invertebrates (144), challenged the concept of a central role of nidogens in BM assembly and instead suggested functional redundancy.

Perlecan HS (from EHS tumor) and heparin (a proxy for the HS chains of proteoglycans) bind to laminins and collagen-IV (120, 122, 145, 146, 147), making HSPGs (119) candidates for providing compensatory linkages. First, HS chains of perlecan and agrin, attached to proteoglycan cores anchored to the laminin layer, are thought to bind to the collagen-IV matrix (106, 148). Second, the HS chains of either HSPG might directly form bridges between laminin and collagen-IV solely through sulfated polysaccharide interactions.

While all BMs contain at least one laminin, nidogen, collagen-IV, and HSPG, they vary in subunit compositions, receptor-interactions, ratios among components, and presence of specialized structures. In a study of the double-nidogen null in the skin, a normal BM architecture at the dermal-epidermal junction, containing laminins, collagen-IV, and perlecan was seen along with a normal skin morphology. On the other hand, capillary BMs showed a greatly reduced distribution of collagen-IV, perlecan, and Lm411, suggesting a key nidogen role for linkage (149). An immunolabeling study of fractionated epidermal BM extracts implied that perlecan rather than nidogens linked the α3/α5 laminins and collagen-IV matrices such that the perlecan core is laminin layer-associated and the HS chains collagen-associated (148). However, perlecan is unlikely to be the sole HSPG mediating linkage since perlecan-deficient keratinocytes did not affect the BM layer in engineered skin (150).

## Cryo-EM structure of the laminin polymer node

**Figure 5:**
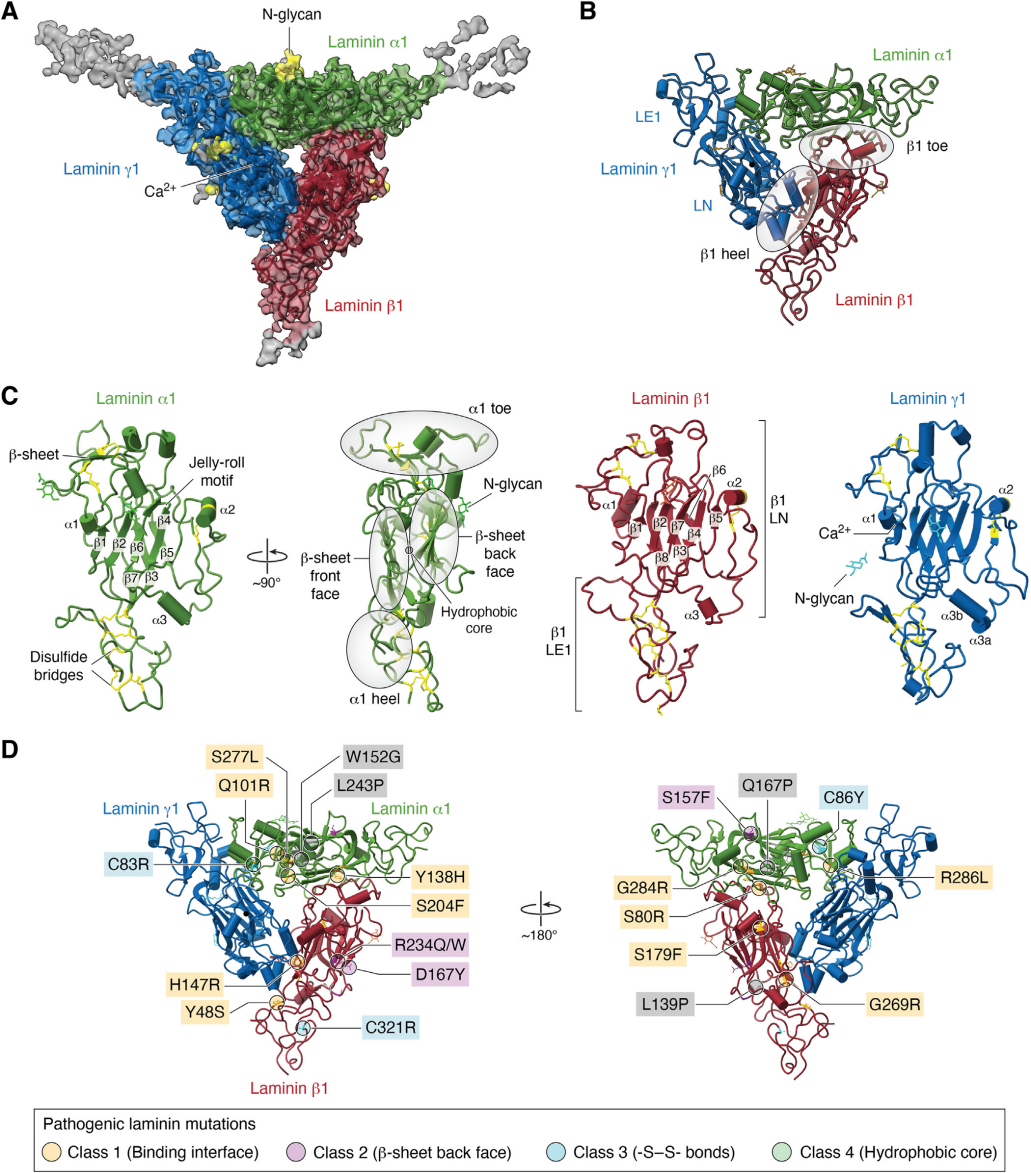
**A cryo-EM structure of the laminin polymer node.***A*, the cryo-EM map (EMD-27542) is color-coded. Laminin subunits α1, β1 and γ1 are shown in *green*, *red* and *blue*, respectively. Flexible parts of the Coulomb map with no model built are shown in *gray*. The N-glycans are colored in *yellow*, and a calcium ion is shown as a *black sphere*. *B*, a cartoon representation of the laminin polymer node structure (PDB ID: 8DMK). The LN and LE1 domains are labeled in γ1 subunit, whereas the toe and heel regions are highlighted in β1 subunit. *C*, atomic models of α1 (shown in *green*), β1 (displayed in *red*), and γ1 (colored in *gray*). Different elements constituting the structures are labeled in the figure. Disulfide bridges are displayed in *yellow*. *D*, pathogenic laminin mutations implicated in LN-lamininopathies. Twenty-three reported to date pathogenic mutations are mapped onto the cryo-EM structure of the laminin polymer node. The amino acid alternations cluster into four distinct classes. The class 1 involve residues from inter-subunit interfaces. The class 2 consists of mutations located in close proximity to invariant N-glycosylation sites. The mutations from class 3 and class 4 affect formation of disulfide bridges and hydrophobic cores of altered laminin variants, respectively.

Recent advances in cryo-EM provide unprecedented insight into structures of dynamic macromolecular complexes at atomic resolution (151, 152, 153, 154, 155). We previously employed cryo-EM to determine a 3.7 Å structure of the laminin polymer node containing the N-terminal 56 kDa α1, 64 kDa β1, and 52 kDa γ1 fragments (Fig. 5*A*) (83, 156). The trimeric laminin complex resembles a triskelion with centrally located LN domains, each consisting of the seven or eight-stranded jelly-roll β-sheet motif, and three rod-like structures projecting outwards, each constituted by one or two EGF-like folds containing LE domains (LEa1 and LEa2) (Fig. 5*B*). Principal Component Analysis of the cryo-EM data revealed the planar and rotational mobility of LE rods, the dynamic property inevitably important for the physicochemical characteristics of the assembled laminin matrix. Due to the intrinsic flexibility of LE rods, additional LE domains present in the analyzed complex were not resolved in the cryo-EM structure. Structural details of individual laminin subunits are depicted in Figure 5*C*. Each individual laminin subunit N-terminal segment has a foot-like shape, also seen in negatively stained images, with the heel formed by LEa1 and the toe consisting of the tip of the LN domain, both regions stabilized by a network of intra-subunit disulfide bonds (Fig. 5*C*).

The LE1a1 domains from α1, β1, and γ1 pack against the outer surfaces of β-sheets from LN domains of neighboring subunits, creating a bend ranging from 110 degrees to 130 degrees between these correspondent LN domains and LE rods (Fig. 5*A*). Inter-subunit interfaces in the complex are formed by two sets of interacting regions. The first set involves loops connecting β1 and β2 strands in β-sheets from neighboring subunits, whereas the second set of interactions involves loops linking strands β7 and β8 from one subunit, and the N-terminal regions along with one of the loops from the LE1 domains of the adjacent subunits.

The inter-subunit interface involving α1 and γ1 within the trimer differs from the α1-β1 and β1-γ1 interfaces, as it is mainly electrostatic in nature and it involves a calcium-binding site from γ1 (83), the latter fact with implications for the mechanism of sequential assembly of laminin trimers into the lattice on cell surfaces. The cryo-EM structure revealed that the α1-γ1 interface involves two loops one in α1, and another loop from γ1. The loop from γ1 consists of amino acids critical for the coordination of a calcium ion, for instance, D108 and T116 (83). We therefore proposed that in the absence of a calcium ion, the loop in γ1 is not structured, hence the α1–γ1 interface cannot be formed, explaining the calcium dependence for the sequential assembly of a trimeric laminin polymer node.

The cryo-EM structure revealed the presence of eight extended densities attached to its surface. The follow-up mass spectrometry glycopeptide analysis (83) demonstrated that these densities represent unique N-glycans dressing the surface of the complex. In addition, the cryo-EM structure reconciled previously obtained site-directed mutagenesis results of inter-subunit interfaces followed by SEC analysis of laminin oligomers reconstituted with genetically-altered subunits. Fifteen of these previously reported amino acid alternations (82) can be mapped onto the inter-subunit binding interfaces. For example, the following mutations resulting in disruption of Lm trimers (82) can be located to these regions in α1 (Y128R, E203R, R263D), β1 (S68R, S200R, E204R, R208E), and in γ1 subunit (Y147R, S213R, D261R). In the majority of cases the aforementioned amino-acid alternations disrupt the network of hydrogen bonds stabilizing the neighboring subunits in the trimeric complex.

## Structural probing of laminin interactions with BM components

**Figure 6:**
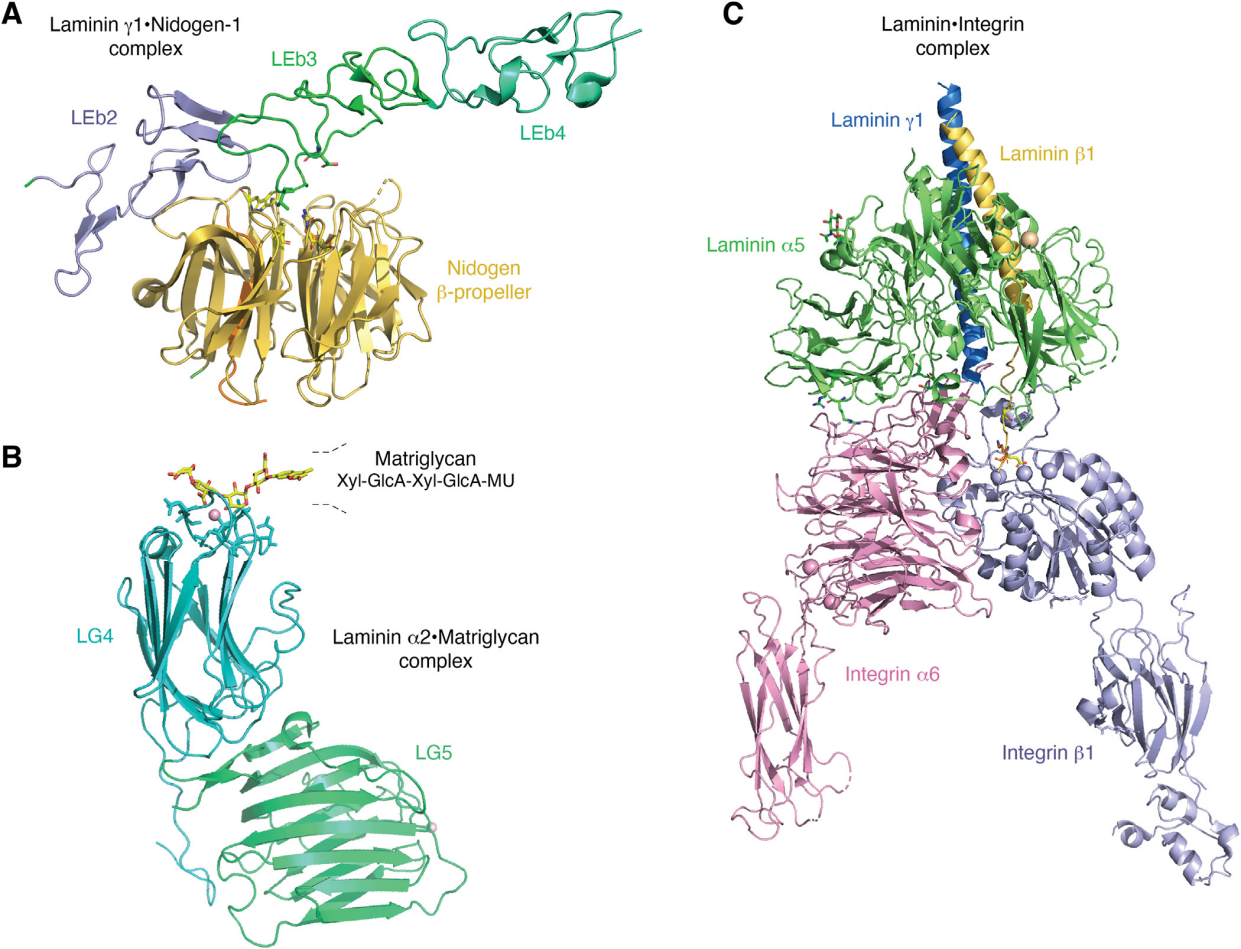
**Structures of other laminin fragments complexed with various BM components.***A*, complex formed between the six-bladed tyr-trp-thr-asp nidogen-1 G3 propeller domain and the laminin γ1-LEb EGF-like domains. The dissociation constant is <1 nM and requires Lmα1N802. We have found that the flanking Ty and EGF6 domains contribute to high-affinity binding as well. *B*, complex formed between the Xyl-GlcA-Xyl-GlcA portion of matriglycan and basic residues of the laminin α2 LG4 domain. A second matriglycan binding site exists in the LG1-3 domains and there are data to suggest that high affinities are seen with LG domain duplication. *C*, a combination of X-ray crystallography and cryo-EM was used to solve the structure of a laminin-integrin complex, critical for BM structure and functions. The interaction requires the α-subunit LG1-3, terminus of the coiled-coil (α-β-γ subunits) binds to the integrin α6 and β1 subunits, involving five different protein domains The C-terminal segment provides two carboxylate anchor points to bridge the metal-ion dependent adhesion locus of the β1 integrin subunit and N189 of α6.

Several structures of individual laminin domains complexed with protein fragments from their BM-binding partners and membrane receptors have been determined using X-ray crystallography and cryo-EM. One such structure is the crystal structure of the nidogen-1 G3-III fragment bound to the laminin segment containing three LE domains from γ1 subunit (LE3-5) (Fig. 6*A*) (157). Nidogen, in addition to interacting with perlecan, acts by linking the laminin lattice with the parallel collagen IV matrix, thus integrating the two protein networks into a functional BM. The structure revealed that laminin binding to nidogen is mediated by LE3 and LE4 domains with its D800, N802 and V804 playing a crucial role in contributing to this nanomolar K_D_ interaction. The structure of LE3–5 bound to nidogen is similar to previously solved X-ray structures of the uncomplexed LE3–5 module (158, 159). The nidogen G3 domain adopts the β-propeller fold with the pseudo-6-fold symmetry consisting of six blades, each formed by the four stranded antiparallel β-sheet. The interaction with laminin is mediated by a number of sidechains lining the upper surface of the central part of the β-propeller.

Another BM interaction trapped in the crystal is the structure of matriglycan complexed with a tandem of the C-terminal laminin LG4-5 domains from α2 chain (160) (Fig. 6*B*). Matriglycan is an O-linked polysaccharide with the following chemical composition ([-GlcA-β1,3-Xyl-α1,3-]n) that is synthesized by the bifunctional glycosyltransferase LARGE (with xylosyl- and gluuronly-transferase activities) (161), and attached to α-DG. The α-DG, in turn, is an extracellular matrix receptor with essential functions in skeletal muscle and the nervous system that acts by anchoring BM to the cell surface through the interactions involving agrin, perlecan, and α laminins. Laminins α associate with cell surface receptors through five tandem LG domains located at their C-termini, with LG4-5 predominantly binding to α-DG. The X-ray structure shows that each LG domain, consisting of approximately 200 amino acids, is folded into a β-sandwich. Overall, the fold of LG4-5 in the structure of the complex resembles the one previously visualized by the structure of a free LG4-5 from α1 chain (62, 162). Each LG domain contains a divalent calcium ion coordinated by residues from one of the rims of the β-sandwich, the fact important for the interaction with the glycosylated α-DG. In the X-ray structure of the matriglycan-LG4-5 complex, a single glucuronic acid-β1,3-xylose disaccharide binds to only one of the LG domains, namely LG4, in a shallow depression centered around the calcium-binding site. Interestingly, the structure revealed that two oxygens, each from one of the carbohydrates, are involved in coordination of the calcium ion with a geometry resembling an octahedron, in which sugar atoms replace two calcium-bound water molecules associated with the protein surface in the absence of the carbohydrate ligand. This chelating binding mode accounts for the high affinity of LG4 interaction with a matriglycan pentasaccharide with the binding constant of 230 nM measured in the presence of calcium.

Yet another example of the interaction involving laminins and other BM components is a recently determined cryo-EM structure of the C-terminal E8 region from laminin 511 composed of a short coiled-coil fragment bundling α5, β1 and γ1 subunits, and the three LG domains (LG1-3) from α5, bound to the prototypic laminin receptor α6β1 integrin headpiece (52) (Fig. 6*C*). Integrins are important heterodimeric receptors that mediate the epithelial cell adhesion to BM. They are composed of a varying number of α and β subunits. In α6β1 headpiece, the α6 subunit consists of the β-propeller and thigh domains, the former fragment interacting with β1 subunit through its βI domain that, in turn, is linked to the C-terminal hybrid and the PSI domains. In addition, the cryo-EM structure consists of two stabilizing Fv-clasp antibodies (not shown), namely TS2/16 binding to the βI domain, and HUTS-4 associated with the hybrid and the PSI domains of β1.

Despite differences in their structural architecture, the prevailing concept is that integrins lacking an αI domain follow a similar activation mechanism incurred upon ligand binding (163). In brief, the ligand provides the carboxylate moiety that directly participates in the coordination of the metal ion chelated by the conserved site on the surface of β subunits, a so-called Metal-Ion-Dependent Adhesion Site (MIDAS) (164, 165, 166, 167). The ligand binding triggers a change in the coordination geometry of MIDAS, subsequently leading to a shift of the α1 and α7 helices from the βI domain and the resultant large displacement of the β hybrid domain that rotates outwards from the β-propeller by an angle of more than 60 degrees (168). These conformational changes of the integrin receptor are then transduced through the cell membrane and coupled with remodeling of the cell’s cytoskeleton (169, 170).

The cryo-EM structure of the aforementioned complex revealed the extensive laminin-integrin interface with E1607 from the C-terminal tail of γ1 involved in the coordination of a metal ion associated with the βI domain, and γ1’s P1609 engaged in binding to the β-propeller of the α6 subunit. In addition, α5 binds to the X1 region of the β-propeller domain through a series of electrostatic interactions; the binding mode that appears to play an important role in ligand capture by the receptor. The comparison of the α6β1 integrin headpiece trapped in the cryo-EM structure of the laminin-integrin complex with the X-ray structure of the unbound α6β1 integrin revealed that while the α6 subunit displays no significant structural rearrangements, the β1 subunit undergoes substantial conformational changes upon ligand binding.

These structural rearrangements are consistent with the already reported conformational changes that were described in the previous paragraph. They include shifting the α1 helix toward MIDAS and the downward movement of α7 helix, which in turn triggers the displacement of the hybrid/PSI domains, collectively switching the receptor from closed to open conformation This closed-to-open transition confirms that the binding of Lm511 to α6β1 integrin follows the general mechanism described for other ligand-integrin interactions.

## Laminins in vertebrate embryogenesis

### Early embryogenesis

The earliest laminin subunits expressed are α1, β1, γ1 and α5 (171). The first BMs formed are those of the parietal and visceral endoderm. Targeted inactivation of LamC1 (Lmγ1 subunit) resulted in embryonic lethality by E5.5 (84). The embryos lacked BMs. The primitive endoderm remained in the inner cell mass with a failure of yolk sac development. Genetic ablation of *LamB1* (Lmβ1 subunit) similarly resulted in embryonic lethality by E5.5 (86). Genetic ablation of *LamA1* (Lmα1 subunit), on the other hand, resulted in lethality at E6.5 (172). In the absence of Lmα1 the endoderm/epiblast BM still formed because of the presence of Lmα5; however, Reichert’s membrane (one of the earliest embryonic BMs) failed to form followed by embryonic death by ∼E6.5. The laminin α5 subunit is present in many BMs from embryos to adulthood.

An analysis of laminin γ1-null embryoid bodies (EBs) revealed that EBs are unable to cavitate or form an epiblast epithelium (173). The addition of laminin to the culture medium of laminin γ1-null EBs was found to restore EB differentiation. However, when laminin was incubated with laminin polymerization-inhibiting fragments E1’ and E4 and the αDG-binding fragment E3, BM assembly and epiblast differentiation was prevented (85). Thus, it was concluded that BM assembly and differentiation in EBs depend upon laminin polymerization in conjunction with cell anchorage through LG domain receptor interactions. A later EB study revealed that Lm111 initiated epithelial cell polarization in which the basal surface was specified by the adherent laminin. An interior laminin injection was found to flip the epithelial cell polarity of the EB (88).

### Late embryogenesis

Despite its early and widespread expression, the absence of the Lmα5 subunit does not cause recognized defects until the later stages of embryogenesis. This is likely due in many tissues to co-expression of compensatory Lmα1 which shares many binding characteristics (174). However, in vasculature, where Lmα4 and possibly Lm3B (but not Lmα1) expression is seen, compensation may be more complex. Overall, Lmα5-null embryos exhibit multiple defects that include failure of anterior neural tube closure, failure of digit septation, and dysmorphogenesis of the placental labyrinth (174).

Lmβ2 expression, on the other hand, is largely confined to the kidney glomerulus, neuromuscular junction, brain, and eye (175, 176, 177, 178, 179, 180). During kidney glomerular capillary loop development there is a switch from Lmα1 to Lmα5 and Lmβ1 to Lmβ2. In the absence of Lmα5, the tuft of endothelial and mesangial cells are extruded from the glomerulus, leading to an absence of vascularized glomeruli at ∼E16.5 in the mouse (181). In the absence of Lmβ2 in kidney, massive proteinuria (mainly albumin) develops despite a normal-appearing glomerulus BM (GBM). However, compensatory Lmβ1 and podocyte foot process fusions were present (182). Interestingly, proteinuria precedes podocyte effacement by about a week, suggesting that the GBM is an albumin barrier that requires Lmβ2 (183). Absence of Lmβ2 also results neuromuscular junctions with a lack of active zones, Schwann cell intrusions into the synaptic cleft and paucity of junctional folds (184). Further, lack of Lmβ2 also results in developmental eye abnormalities that include retinal defects (185). In humans, loss of Lmβ2 causes Pierson syndrome, described ahead.

Lmα4 is found in the vasculature and peripheral nerve. Inactivation of the laminin α4 gene in mice results in hemorrhages during the embryonic and neonatal period and *in vitro* results in deterioration of microvessel growth (186). It is thought that the later expression of vascular Lmα5 limits the period of hemorrhage. Importantly, Lmα4, like Lmα2, is required for normal peripheral nerve myelination (see below) (187).

## Lamininopathies and LN-lamininopathies

Recessive mutations that reduce or ablate laminin subunit expression and function result in diseases collectively called lamininopathies. These include mutations of LAMA2, LAMB2, LAMA1 and LAMA5. The most common and best understood of these are mutations of LAMA2 and LAMB2. Pathogenic mutations that selectively alter the LN domains to reduce or ablate polymerization produce milder diseases that we call LN-lamininopathies (107, 188).

### LAMA2-related dystrophies (LAMA2-RD, MDC1A)

Congenital (CMD) and limb-girdle (LGMD) muscular dystrophies are a group are inherited autosomal recessive diseases affecting skeletal muscle that are often accompanied by defects of peripheral nerve, eye, and brain. These defects result from the absence of or defects in structural components of the ECM and ECM receptors. These defects cause the loss of inter-component bonds needed for ECM structural integrity, the linkage between ECM and cytoskeleton, and transmembrane signaling (83, 156, 189, 190, 191, 192, 193).

Pathogenic variants within the *LAMA2* gene coding for the Lmα2-subunit cause an estimated 30% of cases of congenital muscular dystrophy (190) with over 1000 cases recorded in the Leiden Open Variation Data Bank (194). The pathology consists of muscle degeneration, regeneration from satellite cells, chronic inflammation, and fibrosis. Sensorimotor demyelinating polyneuropathy often accompanies muscle abnormalities. Analysis of mouse models has revealed the neuropathy results from amyelination. During radial axonal processing during peripheral nerve development, Schwann cell (SC) precursor cells extend lamellipodial processes that intercalate between naked axons and envelop them, and then myelinate the larger caliber axons (195). The process requires the cell-polarizing influence of laminin (196, 197). LAMA2-RD is also accompanied by white matter brain anomalies detected by magnetic resonance imaging (MRI) as an augmented white matter signal reflecting increased water content (198).

Patients who are null for *LAMA2* never ambulate and often die of muscle wasting and respiratory failure by the second decade. Patients with reduced and/or defective α2 laminin typically present later in life with prominent weakness but become ambulatory and live longer (199). Currently, there is no cure for either. An estimated 18 to 25% of patients with LAMA2 deficiency have reduced or near-normal laminin by muscle immunostaining (200, 201, 202, 203, 204). A large cluster of the pathogenic mutations causing these reductions map to the Lmα2 LN polymerization domain region (199, 202, 205, 206). These include such mutations as LAMA2- L243P, Q167P, and C73R.

Therapeutic approaches to ameliorate LAMA2-RD have been directed at repairing the transmembrane link between cytoskeleton and BM as well as by inhibiting the sequelae of apoptosis, inflammation, fibrosis, and protein degradation. Drug therapies to treat the sequelae of dystrophy show improvements in animal models, but so far are of limited clinical benefit and importantly do not correct the underlying structural defect (207, 208, 209, 210, 211). While exon-skipping to correct out-of-frame mutations has been used successfully to treat dystrophin-deficiency (Duchenne dystrophy), it seems problematic for laminin-deficiency in that skipping of nearly all *LAMA2* exons will result in either cysteine mispairing with improper domain folding and disulfide-bond formation and/or major loss of functional domains. Recently, two genetic approaches that repair structure have been evaluated in mouse models.

Adeno-associated virus (AAV) is one of the most promising of the somatic gene delivery systems in which high expression can be achieved in muscle and other tissues (212, 213, 214, 215, 216). The experience of human clinical trials with AAV to correct muscular dystrophies has been encouraging. While protein can be lost due to host cellular immune responses to transgene products and AAV capsid (216), this problem has been reduced by avoiding the creation of transgene neoantigens, optimizing serotype, and by adding immunosuppressive therapy (217). Promising success has been recently achieved in the treatment of spinal muscle atrophy (218) and limb-girdle dystrophy type 2D (219). A general limitation, however, is that the DNA coding-insert with promoter, polyA tail, and other elements cannot exceed ∼5 kb (220).

One approach has been to employ AAV serotype 9 (AAV_9_) to deliver small genes coding for two different types of laminin-binding linker proteins (221). One gene codes for αLNNdΔG2’, a chimeric protein consisting of α1 domains LN-LEa fused to the nidogen-1 G3 complex (Ty-propeller-EGF6 domains) providing polymerization activity when bound to a laminin lacking a functional αLN domain (92). The other, miniagrin, links the laminin coiled-coil to αDG (222). The two proteins can bind to Lm411 that is expressed in compensation in the absence of α2 laminins in muscle and nerve (90). In the absence of αLNNdΔG2’ and miniagrin, Lm411 can neither polymerize nor bind αDG. Initial results in laminin-deficient mice, *i.e.*, *dy*^*2J*^*/dy*^*2J*^, *dy*^*W*^*/dy*^*W*^ and *Lama2*−/− *dy*^*3K*^*/dy*^*3K*^ have shown promise (92, 223).

Another approach has been to use AAV to deliver CRISPR-dCAS to cells to activate the *LAMA1* gene, generating Lm111 to replace Lm211 (224). The approach was based on earlier studies that revealed universal transgenic expression of the laminin α1 subunit largely restored normal function and histology in the absence of the laminin α2 subunit (225, 226, 227). The AAV gene activation approach, evaluated in *dy*^*2J*^*/dy*^*2J*^ mice, improved the muscle and peripheral nerve phenotypes and should be effective in the more severely affected animal models (224).

### Pierson syndrome

Pierson syndrome is a congenital nephropathy (and eye disease) caused by mutations in the *LAMB2* gene that alter GBM Lm521 (228, 229). Most of the mutations cause a failure of β2 subunit expression, and the result is severe renal failure. However, several of the missense mutations map to the β2 LN domain and cause a milder disease. Most of these mutations are polymerization defects (91). The Del44 mouse is a new model for Pierson syndrome (massive albuminuria, GBM thickening, glomerulosclerosis, and interstitial fibrosis) resulting from an Lmβ2LN polymerization domain deletion. It causes a 90% reduction in polymerization (230).

A mouse with an amino acid substitution in the Lmα5 LN domain, also preventing polymerization, provides a model for a complex of renal and other defects (231).

## AlphaFold2 modeling of pathogenic laminin polymer nodes

Recently introduced novel artificial intelligence (AI) algorithms have proved to be advantageous in their applications for the modeling of protein structures (156). In particular, the program AlphaFold2 (AF2) (232) employing the transformer neural network to process information extracted from multiple sequence alignments, has demonstrated an unprecedented level of accuracy in predicting some protein structures with sub-angstrom precision. Because laminins are evolutionarily highly conserved proteins that share significant sequence identity and structure homology, we recently utilized AF2 to model structures of 55 monomeric and trimeric laminin complexes, including structures of 23 altered polymer nodes implicated in human disease (https://modelarchive.org/doi/10.5452/ma-kul-lams) (156). It was demonstrated in a quantitative fashion that the *in silico* models display remarkable accuracy and precision by validating a subset of the computed atomic models against experimentally derived cryo-EM structure of the trimeric polymer node (83) and X-ray structures (80, 81) of monomeric laminin subunits. Thus, AF2 is a viable tool for structure modeling and prediction of pathogenic Lm polymer nodes.

Comprehensive and systematic structural analysis of 23 AF2 models of pathogenic laminin complexes consisting of reported to date mutations underlying LN-lamininopathies (Fig. 5*D*) revealed the detailed mechanistic insight explaining how these mutations locally affect protein structures ultimately leading to failures in assembly of laminin polymer nodes (156). Although laminin mutations manifest in a wide spectrum of human diseases, they can be grouped into four distinct classes reflecting their structural roles. Consequently, LN-lamininopathies were re-categorized into four classes based on the underlying molecular basis, rather than associated clinical onsets. Class 1 mutations affect formation of the inter-subunit binding interfaces in Lm polymer nodes Lm1(Q94R)11, Lm2(Y138H)11, Lm2(S204F)11, Lm2(S277L)11, Lm2(G284R)11, Lm21(G269R)1, Lm52(Y48S)1, Lm52(S80R)1, Lm52(H147R)1, Lm52(S179F)1, and Lm5(R286L)21. Amino acid alternations from class 2, which are located in close proximity to the N-glycosylation sites of Lm2(S157F)11, Lm52(D167Y)1, Lm52(R246Q)1 and Lm52(R246W)1, destabilize the local protein conformation and may interfere with N-glycosylation of laminin subunits. Class 3 mutations reported in Lm2(C83R)11, Lm2(C86Y)11, Lm52(C321R)1, and Lm2(C393G)11 destabilize structures of laminin subunits by disrupting specific disulfide bridges. In contrast, amino acid substitutions from class 4 affect formation of hydrophobic cores of Lm2(W152G)11, Lm2(Q167P)11, Lm2(L243P)11 and Lm52(L139P)1. The structural analysis of the AF2 models may foster development of novel therapeutics for the treatment of laminin deficiencies.

## Concluding remarks

Laminins are central to the assembly and physiology of BMs. Thus, the knowledge of the structure-function relationships in this family of proteins is of value for the understanding of a wide spectrum of fundamental concepts in molecular and cellular biology ranging from embryogenesis through intercellular signaling to tissue differentiation and organogenesis. Importantly, the detailed understanding of the roles that laminins play in the formation of ECM has far-reaching implications for the treatment of lamininopathies. The recent advent of structural biology methods, such as cryo-EM (52, 83) and cryo-electron tomography augmented by the state-of-the-art AI algorithms (156), in combination with modern gene therapy/editing technologies (92, 224), and the development of novel mice models, collectively hold the premise for providing a paradigm shift in a foreseeable future for the treatment of lamininopathies by engineering artificial BMs for tissue implants and structure-based drug design. Before this goal is achieved, however, further structural and functional probing of the interactions involving laminins and other BM components, and investigation of the cellular consequences of these interactions, will be needed in the context of the assembled ECM *in vitro* and *in vivo*.

## Conflict of interest

P. D. Y. was an editorial board member for the *Journal of Biological Chemistry* until December 31, 2023, and was not involved in the editorial review or the decision to publish this article. P. D. Y. received royalty payments from Rutgers University as an inventor on patent application entitled “AAV-compatible laminin-linker polymerization proteins” which was licensed to SEAL Therapeutics. A. W. K. has no known competing financial interests or personal relationships that could have appeared to influence the work in this article.
